# Current Developments in Microvolt T-wave Alternans

**Published:** 2006-10-01

**Authors:** Beata Sredniawa, Agata Musialik-Lydka, Jacek Kowalczyk, Zbigniew Kalarus

**Affiliations:** First Department of Cardiology, Silesian Medical School, Silesian Center for Heart Diseases, Zabrze, Poland

**Keywords:** microvolt T wave alternans, implantable cardioverter-defibrillator, ventricular arrhythmias, sudden cardiac death, risk stratification

## Abstract

Microvolt T-wave alternans (MTWA), the beat-to-beat fluctuation in T wave amplitude and morphology, is closely linked to vulnerability to ventricular arrhythmias in various experimental and clinical conditions. Clinically, MTWA is most commonly measured using the spectral method, although non-spectral methods for its assessment from ambulatory electrocardiographical recordings also have been developed. Recent studies suggest that the quantitative assessment of TWA may also be clinically relevant. The standardisation of the criteria for abnormal MTWA test still needs to be completed. The expansion of indications for implantable cardioverter-defibrillator (ICD) therapy following the positive results of the MADIT-II and SCD-HeFT trials might have unacceptable economic and medical consequences, and therefore new tests are needed to better discriminate patients who will and will not benefit from ICD implantation. A recent meta-analysis of  MTWA studies revealed an overall positive predictive value for arrhythmic events of 19.3%, negative predictive value of 97.2%, and 3.77% univariate relative risk of arrhythmic events. The negative predictive value of MTWA in MADIT-II type patients has been reported to be 97.5%. The predictive value of the test varied significantly in different patient population. Current data support the use of MTWA testing for evaluation of patients with low ejection fraction who are considered for ICD implantation. The independence of the prognostic value of MTWA from other clinical and electrophysiological variables needs further confirmation.

## Introduction

Microvolt T-wave alternans (MTWA) represents the phenomenon of alteration in the shape and/or amplitude of the T wave on an every other beat basis in the microvolt (invisible to the naked eye) range. MTWA predisposes towards malignant ventricular tachyarrhythmias in various experimental conditions and clinical disorders and therefore has become in recent years a widely discussed noninvasive index, which can serve as a predictor of sudden cardiac death (SCD) [1]. During the last years, substantial progress has been made in understanding the cellular mechanism of MTWA, refining the methods of its testing and, most importantly, determining its clinical value. Recent studies have provided strong indication that MTWA may be very useful for identifying patients who will and especially those will not benefit from implantable cardioverter-defibrillator (ICD) therapy among all ischaemic heart disease (IHD) patients with low ejection fraction (EF) ("MADIT II-like" patients), thus solving the "The MADIT II Conundrum" [2].

This review concentrates mainly on the most important basic and clinical studies on MTWA published since 2003; those published earlier have been summarised in a previous article[3].

## Basic Research on MTWA

While it is already firmly established that MTWA arises from alternation of repolarisation at the level of the single myocyte [4], the exact cellular mechanism of MTWA is not yet completely clear. The two main hypotheses (which are not mutually exclusive) are that MTWA develops, a) when the myocyte cannot handle intracellular Ca^2+^ during each cardiac cycle, and b) when the slope of the action potential duration (APD) restitution curve is > 1, as determined by the kinetics of the cell membrane currents [4]. Pruvot et al [5]. used high-resolution optical mapping techniques to measure action potentials and Ca^2+^ transients simultaneously from hundreds of epicardial sites in the guinea pig model of pacing-induced TWA. They found that APD and Ca^2+^ alternans were remarkably similar both spatially and temporally and consistently occurred together near the base of the left ventricle, and not where APD restitution was steepest. They concluded that the mechanism of TWA in the intact heart is more closely associated with intracellular Ca^2+^ cycling rather than APD restitution [5].

T wave alternans, which arises as a single cell phenomenon, is not evenly distributed throughout the heart and its magnitude and phase can differ substantially between neighbouring areas (discordant alternans) [4]. Spatially discordant alternans provides a substrate for reentrant ventricular tachyarrhythmias [4].

MTWA is critically related to changes in heart rate. In 2000 Narayan and Smith [6] reported that MTWA was significantly greater and differently distributed within the JT-segment during heart rate deceleration compared to the same heart rates during acceleration (from 100 to 150 beats per minute) induced by atrial and ventricular pacing during electrophysiological (EP) study. The phenomenon was called TWA hysteresis, similarly to the well-known hysteresis of the QT interval following abrupt heart rate changes [7]. In this study [6] the time course of alternans hysteresis and the distribution of alternans within the JT segment were distinctly different between patient with and without inducible arrhythmias. The sensitivity and specificity of alternans for arrhythmia inducibility varied asymmetrically during heart rate acceleration and deceleration, which has important implications for the clinical utility of the test for risk stratification.

Other authors have used the term "alternans memory" instead of "hysteresis" [4], although this can create confusion with the fundamental concept of "cardiac memory".

In a study with isolated guinea pig ventricular myocytes and Langendorff-perfused guinea pig whole hearts, Walker et al. [8] also demonstrated that alternans persisted at heart rates below those that were required to induce it. Their results showed that TWA was an intrinsic property of cardiac myocytes and suggested an important underlying role of calcium cycling in the mechanism of alternans. Moreover, they demonstrated that the hysteresis effect was stronger for the more arrhythmogenic form of TWA, namely discordant alternans, than for the less arrhythmogenic concordant TWA [8].

The hysteresis demonstrates that MTWA (and hence, repolarisation dispersion) depends not only on the heart rate, but also on the direction of change of heart rate (the "heart rate history"). The persistence of MTWA at slower heart rates preceded by transient heart rate perturbations provides a possible explanation for the occurrence of ventricular arrhythmias and SCD at heart rates lower than those necessary to elicit MTWA during clinical testing [8].

Bernus et al. [9] demonstrated in a computer model of acute regional ischemia that ischaemia-related arrhythmias were triggered by calcium-mediated alternating conduction blocks in the ischaemic border zone, which led to intramural reentry and APD alternans. In an experimental model with endocardial and epicardial electrograms recorded in dogs during left anterior coronary artery occlusion and right atrial pacing, Nearing and Verrier observed orderly stepwise progression of the complexity of T wave oscillations (e.g. ABC-ABC or ABCD-ABCD patterns, more complex patterns, etc.), discordant alternans and subsequently progression to VF [10]. This study demonstrated for the first time mechanistic link underlying the ability of TWA to predict malignant arrhythmias.

The relation between MTWA and cardiac sympathetic nervous system abnormality was recently documented with the help of Iodine-123 (I-123) metaiodobenzylguanidine (MIBG) myocardial imaging. In patients with idiopathic cardiomyopathy, MIBG imaging revealed defect areas of sympathetic denervation. In this study, a relation was found between MIBG indices reflecting sympathethic denervation and shifted sympathetic tone [11]. Using MIBG imaging, it has been demonstrated that in patients with non-ischemic cardiomyopathy improvement of EF after long term beta-blockers therapy correlated with improvement in cardiac sympathetic innervation and was associated with decrease in the number  of patients with positive MTWA and the decrease of MTWA voltage [12].

## Development of the Methodology

There are several methods for measurement of MTWA (for comprehensive reviews see [13,14]). The most commonly used is the spectral method, whose principles are described in detail elsewhere [1,3]. To achieve the necessary increase in heart rate, early studies used invasive atrial pacing, whereas nowadays noninvasive exercise stress test with bicycle or treadmill is mainly used. It has been shown that the two methods provide equivalent results [15]. Recently a method for measurement of MTWA from ambulatory ECG (Holter) recordings has been developed. The rationale is to investigate the presence of MTWA in periods when the occurrence of SCD is the most frequent, that is, at peak heart rate, early morning and during peak ST-elevation [1,14]. Since the spectral analytical method requires stationary data, which cannot be acquired with ambulatory ECG monitoring, TWA from Holter recordings is analysed using a non-spectral technique, called the (Modified Moving Average Beat Analysis, MMA) (detailed description of the method can be found in [14]). Verrier et al. [16] analysed the Holter database of the Autonomic Tone and Reflexes after Myocardial Infarction (ATRAMI) study [17] and demonstrated for the first time the value of MTWA acquired from ambulatory ECG recordings for risk stratification for SCD. The value of MMA methods needs to be further tested in prospective studies [14]. According to the statement of Centers for Medicare and Medical Services released in March 2006 [18], "Microvolt T-wave Alternans diagnostic testing is covered for the evaluation of patients at risk for SCD, only when the spectral analysis method is used."

The original definitions and classification criteria of the spectral analysis of MTWA were strictly established [13]:

The test is considered *positive* if it has sustained alternans with an onset heart rate ≤110 beats/min. or has sustained alternans at the resting heart rate, even if the latter is >110 beats/min.
  Sustained alternans is defined as lasting at least 1 minute with alternans voltage (V_alt_) ≥1.9 μV and alternans ratio≥3 (the ratio of the alternans power divided by the standard deviation of the noise in the reference frequency band, a measure of the statistical significance of the alternans), in any of the vector leads X, Y, Z, or VM (the vector magnitude), or in a precordial lead and confirmed (with V_alt_) ≥1.9 μV) in an adjacent precordial lead, with some period of artifact free-data (defined as ectopic or premature beats ≤10% of all beats, no respiratory activity at 0.25 cycles per beat, variation in instantaneous heart rate <30 beats/min and no RR interval alternans present ≥2 msec with alternans ratio≥3).
The MTWA test is *negative* if 1) it does not meet criteria for being positive and 2) maximum negative heart rate is ≥105 beats/min. The maximum negative heart rate is the highest interval heart rate associated with an interval without significant alternans, with noise level in the vector magnitude lead ≤1.8 μV, with ≤10% ectopic beats.
The test is *indeterminate* if it can not be definitively classified as positive and negative. In recent studies, indeterminate test result, which occurs in 9-47% of the results [13], is grouped together with positive test as "abnormal MTWA", as opposed to negative test. This seems appropriate only if indeterminacy reflects ectopy, rather than ECG noise [1,13].

**Figure 1:**
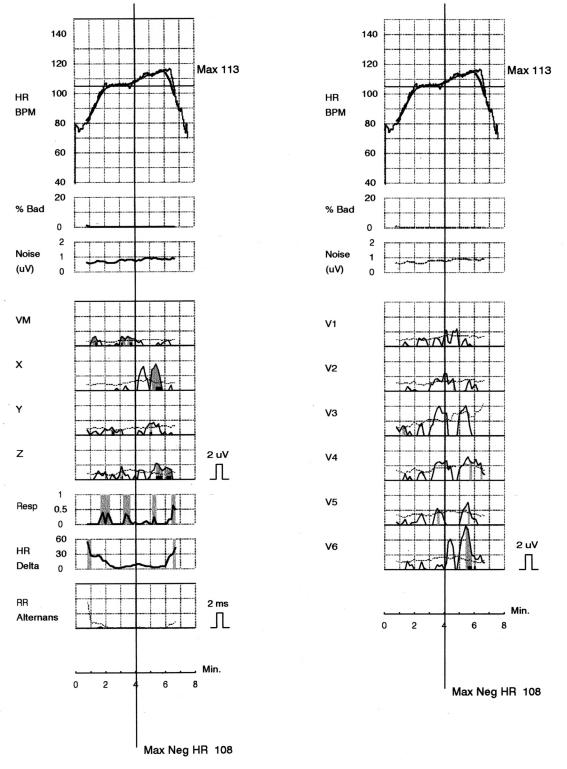
An example of negative microvolt TWA recorded before ICD implantation in ischaemic patient with history of cardiac arrest due to sustained ventricular tachycardia. No ventricular arrhythmic events were documented during 1 year following implantation.

An example of negative MTWA taken from ischaemic patient before ICD impalantation because of secondary prevention without arrhythmic events during 1 year follow-up using Cambridge Heart, Bedford, Massachusetts is presented in Figure 1.

**Figure 2:**
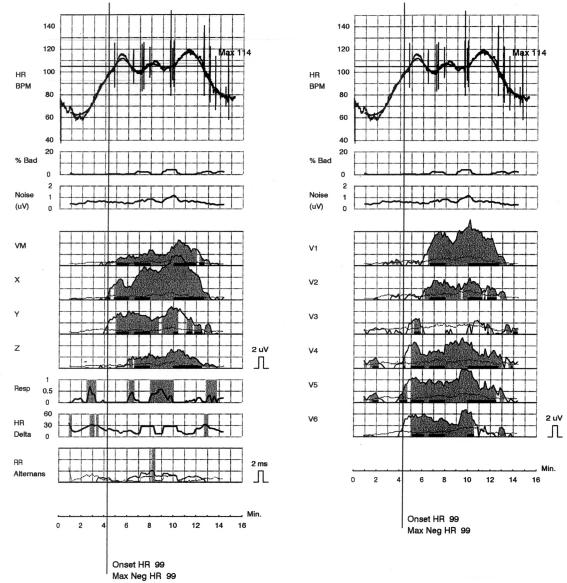
An example of positive microvolt TWA recorded before ICD implantation in a patient with ischaemic cardiomyopathy and history of sustained ventricular tachycardia. The patient suffered several episodes of VT/VF during the third month following implantation.

Figure 2 presents a positive test recorded in a patient with ischaemic cardiomyopathy before ICD implantation due to secondary prevention, who received during the third month of follow-up appropriate shocks because of ventricular fibrillation and sustained ventricular tachycardia (both examples are from our database).

Recently it has been suggested that quantitative assessment of MTWA may provide independent information. Klingenheben et al. [19] reported that MTWA had higher magnitude (V_alt_) and was positive in more ECG leads in cardiomyopathy patients with compared to those without tachyarrhythmias and in patients with non-ischaemic than in those with ischaemic cardiomyopathy. The authors suggested that more extensive MTWA might reflect more extensive myocardial damage and higher arrhythmia propensity [19].

Currently it is accepted to keep beta-blocker agents during TWA testing [14]. It has been documented that beta-blockade diminishes the amplitude of MTWA. Therefore in order to assess the risk of the patient for SCD it is reasonable to continue current medications during the test [21]

In a recent substudy of the Cardiac Arrhythmias and Risk Stratification after Myocardial Infarction (CARISMA) trial [22], three methods of TWA assessment were compared: exercise testing, atrial pacing and simultaneous ventricular and atrial pacing [23]. The authors found 79% concordance between exercise and atrial pacing and 95% between the two pacing mode. Indeterminate alternans was present in 7% during ventricular and atrial pacing and in 24% during exercise. The high concordance between the tests and the low number of indeterminate tests indicate that the ventricular and atrial pacing method can be useful in TWA evaluation in patients who are not able to complete exercise test [23].

## TWA in clinical practice: meta-analysis, era of MADIT-II and SCD-HeFT populations and dilated cardiomyopathy

There is already overwhelming evidence that treatment with ICD significantly reduces mortality in patients with ischaemic heart disease with reduced left ventricular EF. The second Multicenter Automatic Defibrillation Trial (MADIT-II) demonstrated 28% reduction of mortality rates (absolute mortality reduction of 5.6%) during an average follow-up of 20 months in post-myocardial infarction (MI) patients with EF ≤30% in whom ICD was implanted compared to those who received only conventional therapy [24]. In the Sudden Cardiac Death in Heart Failure Trial (SCD-HeFT), which enrolled patients with heart failure of ischaemic or nonischaemic origin in New York Heart Association (NYHA) class II or III and EF ≤35% with a mean followed up of 45.5 months, amiodarone did not improve survival, while single-lead shocked ICD therapy reduced overall mortality by 23% [25]. It is economically unrealistic and ethically questionable to expect all MADIT II-like patients to receive ICD therapy, given the size of this population (55,000 to 65,000 new cases annually in the U.S.A. [26]) and the fact that the majority of these patients will never need the device. Therefore it is mandatory to find methods to differentiate patients who really need ICD implantation among all MADIT II-like patients [27,28]. Moreover, consensus panels accepted the scientific validilty of MADIT-II, but recognized the need for better methods of risk stratification [29]. In this context the recent interest in research, assessment and clinical studies in T-wave alternans is reasonable and justified.

In 2005, Gehi et al. reported a meta-analysis of MTWA studies to clarify the predictive accuracy and practical usefulness of the test [30]. The analysis included 19 prospective studies with a total of 2,608 patients published between January 1900 and December 2004. The average follow-up was 21 months. The population included wide range of subjects: patients with congestive heart failure (CHF), ischemic CHF, non-ischemic, post-MI, athletes and healthy subjects. In the analysis indeterminate MTWA tests were excluded. The arrhythmic event was classified as SCD, cardiac death, VT, VF, or ICD shocks. Summary positive predictive value (PPV) of MTWA for arrhythmic events was 19.3% (95% confidence interval [CI] 17.7% to 21.0%), negative predictive value (NPV) was 97.2% (95% CI 96.5% to 97.9%), and the univariate relative risk (RR) was 3.77 (95% CI 2.39 to 5.95). In patients with arrhythmic history, PPV and NPV were 51.0% and 86.0%, respectively, whereas in those without arrhythmic history they were 15.4% and 98.1%, respectively. There were no differences in PPV, NPV and RR between ischemic and non-ischemic CHF patients, but PPV was higher in CHF patients than in post-MI patients. In 8 studies indeterminate MTWA was included in the definition of abnormal test. Comparison of the results with and without inclusion of indeterminate test in these studies showed no significant differences in the predictive value of an abnormal test. In 3 studies, in which multivariate analysis was performed including MTWA and other tests, such as EF, left ventricular systolic diameter, NSVT, signal-average electrocardiogram and QT dispersion, MTWA was independently predictive of arrhythmic events [3]. Whilst the 97% NPV confirmed that a negative MTWA test can identify patients at low-risk for arrhythmic events (24-54% of the subjects in this analysis), in the decision who should receive an ICD for primary prophylaxis the 19% PPV for arrhythmic events is more relevant. The PPV of MTWA varied (from 0% to 51%) depending on the clinical characteristic of tested patients and the pretest probability for arrhythmic events. This meta-analysis also revealed the need for standardisation of the assessment of the results of MTWA. It seems fundamentally erroneous to define as "abnormal" a test, which is indeterminate because of excessive noise that makes the record uninterpretable [30]. Further prospective studies are needed to define the independent predictive value or MTWA for cardiac arrhythmias in multivariate analysis including many demographical, clinical and electrophysiological variables. In addition, future MTWA trials should provide analysis of all-cause (in addition to arrhythmic) mortality, and should evaluate the mortality reduction benefit of implantable cardioverter-defibrillators (ICDs) in patients who test MTWA-negative and non- negative [31].

Four recently published major studies assessed MTWA in MADIT-II like patients, and patients with EF ≤35% or ≤40%. In the first one, included in the meta-analysis, in 129 patients who met MADIT-II criteria, Hohnloser et al. reported no cardiac death or cardiac arrest during 2 years of follow-up in patients with normal TWA, compared to 15.6% among patients with abnormal test [32].

In the next, more recent study, also included in the meta-analysis, on 177 MADIT-II like patients, Bloomfield et al. used MTWA and QRS duration to identify groups at high and low risk of the all-cause mortality (primary end point) during a follow-up of 2 years2. Abnormal (positive and indeterminate) MTWA was found in 68% patients, whereas 32% had QRS>120 ms. The 2-year actuarial mortality rate for patients with abnormal MTWA was significantly greater (17.8%) than in patients with normal MTWA (3.8%, p=0.02, hazard ratio 4.8). It was also significantly lower in patients with normal MTWA test compared to those with narrow QRS (3.8%, 95% CI: 0-9.0 vs 12.0%, 95% CI: 5.6-18.5). The corresponding false-negative rates were of 3.5% and 10.2%, respectively. The authors concluded that in MADIT-II like population, abnormal MTWA was a strong predictor of all-cause mortality and identified better that QRS duration patients who were not likely to benefit from ICD therapy [2].

In a more recent report, the same authors extended their initial findings in analysis of 587 patients (half with ischemic heart disease) with EF ≤40%. Of them, 66% patients had an abnormal MTWA test. The mean follow-up was 20±6 months and primary end point was death and non-fatal sustained ventricular arrhythmia. The 2-year actuarial event rate was 15.0% in patients with abnormal MTWA test and 2.5% in those with normal (RR 6.5; 95% CI 2.4 to 18.1). Survival of patients with normal MTWA test was 97.5% [33].

Recently, Chow et al. [27] reported the results of a study on 768 patients with ischaemic cardiomyopathy, EF ≤35% and no arrhythmia history. All patients underwent baseline MTWA test which was classified as negative and non-negative and were followed-up for an average of 18±10 months. After multivariable adjustment, a non-negative MTWA test was associated with a significantly higher risk for all-cause (hazard ratio 2.24 (95% CI: 1.34 to 3.75), p=0.002) and arrhythmic mortality (hazard ratio 2.29 (1.00 to 5.24), p=0.049).  In subgroup analyses, a non-negative MTWA test was also associated with a higher risk for all-cause mortality in patients with EF ≤30% (hazard ratio 2.10 (1.18 to 3.73), p=0.01) and after excluding those with indeterminate MTWA tests (hazard ratio 2.08 (1.18 to 3.66), p=0.01) [27].

The conclusions from the above 4 studies are similar: MTWA testing not only identifies ischemic patients at high risk of arrhythmias, but also those who are not likely to benefit from ICD therapy [27,32,33]. In the MADIT-II trial, 18 ICDs had to be implanted to save one life. ICD therapy diminishes the quality of life or even can be proarrhythmic [34]. Based on the MADIT-II data, incremental cost-effectiveness ratio (iCER) was 235,000$ per year-of-life saved, and in perspective of 12 years this ratio ranged from 78,600 to 114,000$ [26]. High cost, lack of enough hospital and physician resources or the perception that current criteria are too broad limit the access to the ICD for many candidates for this therapy [28]. For these reasons MTWA may be useful as a screening test to identify patients who will not benefit from ICD implantation [2]. Applying this strategy to a MADIT-II likely population, only 7 ICDs would have to be implanted to save 1 life [32]. It has been proposed that if the decision not to implant an ICD would be taken on the basis of a negative MTWA test, it would seem reasonable to repeat the test every 1-2 years, since most prospective studies had follow-up between 1 and 2 years [35].

The ongoing Microvolt T Wave Alternans Testing for Risk Stratification of Post MI Patients (MASTER I) trial [36] is expected to provide new information about the usefulness of MTWA for the risk-stratification of MADIT-II like patients.

MTWA seems to be a promising risk factor for prediction of arrhythmic events in idiopathic dilated cardiomyopathy (DCM). In one observational prospective study, 137 patients with DCM underwent risk stratification using MTWA, EF, baroreflex sensitivity (BRS), heart rate variability, presence of nonsustained ventricular tachycardia (VT), signal-averaged electrocardiogram and presence of intraventricular conduction defect [37]. MTWA and BRS were significant univariate predictors of sudden death, resuscitated VF, or documented hemodynamically unstable VT during an average follow-up of 14±6 months, whereas in multivariate analysis only MTWA was a significant predictor. These results were supported by 2 more studies, one of them on patients with congestive heart failure NYHA class II [38], and the other on patients in NYHA class III [39]. In the former study which enrolled 81 patients, the NPV and PPV of TWA for SCD, documented sustained VT/VF, or appropriate ICD shock were 100% and 24%, respectively [38]. The latter study enrolled 46 patients, of whom 7 patients (16%) died from cardiac death (non-sudden in six and sudden in one) during the follow-up of 1.6 years. MTWA was positive in 24 (52%), negative in 13 (28%) and indeterminate in 9 patients (20%). MTWA was positive in all patients with events (100%), but only in 16 of 37 patients without events (41%) (p=0.02) [39]. On the other hand, in the Marburg Cardiomyopathy Study, which enrolled 343 patients with idiopathic DCM followed-up for 52±21 months, MTWA was not independently predictive for major arrhythmic events (46 patients, 13%) in multivariate analysis including reduced EF, lack of beta-blockers therapy, signal-averaged ECG, BRS, heart rate variability [40].

The ongoing T-wave Alternans in Patients with Heart Failure (ALPHA) trial is designed to evaluate the independent predictive value of MTWA on the combined occurrence of cardiac death and life-threatening arrhythmias, in a population of patients with non-ischemic dilated cardiomyopathy given NYHA class II and III, over 12-24 months follow-up [41].

## MTWA in other diseases and populations

Molon et al. performed MTWA testing in patients with type 2 diabetes mellitus [42] and reported that the test was positive in 21% of diabetic patients without any cardiac disease. MTWA positive patients had significantly higher HbA1c levels than those with negative MTWA. The authors concluded that positive MTWA is correlated with glycaemic control [42].

Kirchof et al. performed MTWA test at rest and during exercise in nine symptomatic, inducible patients with established Brugada syndrome and in seven healthy controls [43].  They concluded that MTWA cannot detect arrhythmia propensity in patients with is not appropriate in Brugada syndrome.

Furlanello et al. [44] followed up 100 competitive athletes (including 72 elite), 48 of them normal and 52 with severe arrhythmias, for 36.1 months. They reported that positive MTWA was connected with symptomatic arrhythmias. During electrophysiological study, sustained monomorphic VT was induced in 72% of patients with positive MTWA. The authors concluded that MTWA seems to be useful in the assessment the risk of arrhythmic events in athletes.

Narayan et al. [45] studied 28 patients with coronary artery disease, systolic dysfunction and nonsustained VT. They found that TWA magnitude, which was computed spectrally during ventricular stimulation, varied linearly with LV mass index (p=0.003). In addition, positive TWA (magnitude ≥1.9 μV) in the orthogonal electrocardiographic axes overlaid scar or wall motion abnormalities in corresponding echocardiographic segments (p<0.05), which suggests that TWA may indicate arrhythmic contributions from regional myocardial scar and eccentric LV hypertrophy.

## Conclusions

MTWA is a non-invasive index which is strongly correlated with arrhythmic events. Due to its very high negative predictive value, MTWA is especially useful as a screening method for identifying patients with low ejection fraction at low risk for arrhythmic events who are very unlikely to benefit from ICD implantation. The interpretation of the test with regard to "normal" and "abnormal" test still needs to be standardised. Quantitative methods of MTWA assessment seem promising and need further investigations.
